# Comparative accuracy of sleep disturbance questionnaires in children with autism spectrum disorder: a receiver operating characteristic study

**DOI:** 10.1136/bmjpo-2025-004097

**Published:** 2026-05-06

**Authors:** Batuhan Yüksel, Mert Dogan, Koray Kara, Ozgun Kaya Kara

**Affiliations:** 1 Department of Physiotherapy and Rehabilitation, Faculty of Health Sciences, Akdeniz University, Antalya, Turkey; 2 Department of Child and Adolescent Psychiatry, University of Health Sciences Turkey, Antalya Training and Research Hospital, Antalya, Turkey

**Keywords:** Sleep, Child Health, Child Psychiatry, Rehabilitation

## Abstract

**Objective:**

The aim of this study was to investigate the comparative effectiveness of various sleep and wakefulness scales used in children and their usefulness for children with autism spectrum disorder (ASD).

**Design:**

Cross-sectional observational study.

**Setting:**

The study was conducted at the Department of Child and Adolescent Psychiatry, University of Health Sciences Research and Training Hospital, Antalya, Türkiye, which serves as a regional referral centre for children and adolescents with developmental and behavioural disorders.

**Participants:**

A total of 230 children participated in the study, including 155 children diagnosed with ASD and 75 typically developing peers, aged between 4 and 18 years.

**Primary outcome measures:**

Sleep-wake disorders were assessed using five questionnaires: the Children’s Sleep Habits Questionnaire (CSHQ), Epworth Sleepiness Scale for Children and Adolescents (ESS-CHAD), Paediatric Daytime Sleepiness Scale (PDSS), Paediatric Sleep Questionnaire (PSQ) and Sleep Disturbance Scale for Children (SDSC). These tools evaluated sleep patterns, daytime sleepiness, and sleep-related behavioural problems based on caregiver reports.

**Results:**

The mean age of children with ASD and the typically developing peer group was 9.0±3.69 years and 9.07±4.25 years respectively. The CSHQ, ESS-CHAD, PDSS, PSQ and SDSC all demonstrated discriminant validity and internal consistency in the assessment of sleep-wake disorders in children with ASD (p<0.05, Cronbach’s alpha >0.60). The CSHQ and SDSC showed similar levels of diagnostic accuracy in detecting sleep disorders. The PSQ had a high positive predictive value, the PDSS demonstrated a high negative predictive value and the ESS-CHAD showed a high level of agreement.

**Conclusions:**

A combination of tools such as the CSHQ, ESS-CHAD, PDSS, PSQ and SDSC may be useful for assessing sleep-wake disorders in children with ASD.

**WHAT IS ALREADY KNOWN ON THIS TOPIC**

- Sleep disturbances affect up to 80% of children with autism spectrum disorder (ASD), yet no head-to-head comparison of available screening questionnaires has been conducted in this population.

**WHAT THIS STUDY ADDS**

- Using receiver operating characteristic analysis in 155 children with ASD, this study demonstrates that the Children’s Sleep Habits Questionnaire (CSHQ) and Sleep Disturbance Scale for Children (SDSC) provide the highest diagnostic accuracy, while the Epworth Sleepiness Scale for Children and Adolescents (ESS-CHAD) and Paediatric Daytime Sleepiness Scale (PDSS) offer viable rapid-screening alternatives.

**HOW THIS STUDY MIGHT AFFECT RESEARCH, PRACTICE OR POLICY**

- These findings support a tiered instrument-selection approach—prioritising the CSHQ or SDSC for comprehensive screening and the ESS-CHAD or PDSS for time-limited clinical settings—to standardise sleep assessment in paediatric ASD care.

## Introduction

 Autism spectrum disorder (ASD) is a long-term neurodevelopmental condition that commonly emerges in early childhood and results in difficulties in social communication and relationship formation.^1^ According to the WHO, ASD affects approximately one in every 160 children. The primary diagnostic criteria for ASD, as outlined in the Diagnostic and Statistical Manual of Mental Disorders (DSM) V, include impairments in social communication and interaction, and restricted and repetitive patterns of interests, behaviours and activities.^1^ It has also been estimated that–50%–80% of children with ASD experience sleep difficulties during preschool and school years.^2 3^

Common sleep problems observed in children with ASD include extended time to fall asleep, inconsistent sleep patterns, difficulty initiating sleep at night, resistance to going to bed, delays in the body’s natural sleep-wake cycle, reduced overall sleep duration, frequent awakenings at night, daytime sleepiness, reduced sleep quality and rapid eye movement (REM) disorder.^4^ These sleep problems can have significant negative effects on children’s behaviour, attention, academic performance and social functioning. Insufficient sleep can lead to increased impulsivity and behavioural issues, decreased attention, lower academic performance and impaired social functioning. Therefore, it is important to address sleep problems in children with ASD as they can worsen core symptoms and contribute to increased cognitive impairment.^5 6^ Parents of children with ASD should be particularly attentive to sleep issues from early childhood to adolescence.^7^

The likelihood of a child experiencing sleep problems is heightened when several factors affect sleep work in concert. Moreover, sleep problems can exacerbate each other, making it crucial to understand these issues. Researchers have developed numerous questionnaires to investigate sleep habits and problems.^8^,^10^ These tools were designed to shed light on the complexities of sleep and the challenges faced by children. In the clinic, the use of scales based on family self-reports provides useful information on sleep habits and the effects of sleep problems on daily life in a practical way. In the current study, the Children’s Sleep Habits Questionnaire (CSHQ), Epworth Sleepiness Scale for Children and Adolescents (ESS-CHAD), Paediatric Daytime Sleepiness Scale (PDSS), Paediatric Sleep Questionnaire (PSQ) and Sleep Disturbance Scale for Children (SDSC) scales were used to evaluate sleep problems. These questionnaires were selected because they assess complementary dimensions of sleep and wakefulness in paediatric populations. The CSHQ primarily evaluates behavioural sleep habits and sleep-related difficulties, the ESS-CHAD and PDSS focus on daytime sleepiness, the PSQ assesses sleep-related breathing symptoms and associated behavioural problems, and the SDSC provides a multidimensional assessment of different domains of sleep disturbances. Most previous studies have focused on the validation or psychometric properties of individual instruments rather than evaluating their relative diagnostic performance.^11^,^14^ In addition, comparative evidence regarding the discriminatory performance of commonly used sleep questionnaires remains limited in Turkish paediatric populations, particularly across the wide developmental age range of 4–18 years. Therefore, the aim of this study was to investigate the comparative effectiveness of several widely used sleep and wakefulness scales in children with ASD.

## Methods

This study was conducted in the Department of Child and Adolescent Psychiatry at the University of Health Sciences Research and Training Hospital, Antalya, Türkiye, which serves as a regional referral centre for children and adolescents with developmental and behavioural disorders. Participants were recruited from children presenting to this clinic during the study period who met the eligibility criteria.

### Participants

This cross-sectional study included a total of 155 children diagnosed with ASD (131 males, 24 females) with a mean age of 9.0±3.69 years, and a control group of 75 typically developing children (37 males, 38 females) with a mean age of 10.2±4.25 years.

The eligibility criteria for children with ASD were as follows: (1) a confirmed diagnosis of ASD by a child and adolescent psychiatrist according to DSM-5 criteria, (2) an age range of 4–18 years and (3) willingness to participate in the study. Children with comorbid neurological or genetic disorders that could independently affect sleep patterns (eg, cerebral palsy or genetic syndromes) were excluded. Information regarding intellectual disability and medication use was obtained from medical records and caregiver reports. The inclusion criteria for typically developing children were: (1) no diagnosis of neurological or psychiatric disorders and (2) age between 4 and 18 years. The exclusion criteria for typically developing children were (1) not willing to participate in the study and (2) the presence of any sleep problems, as determined based on caregiver reports obtained during the screening process and review of medical history.

### Outcome measures

The sociodemographic form was completed by the researchers and information was retrieved from the medical records of the patients. The Turkish versions of the CSHQ,^15^ ESS-CHAD,^16^ PDSS,^17^ PSQ^18^ and SDSC^19^ were completed by the primary caregivers of the children with ASD and their typically developing peers under the supervision of researchers.

#### Children’s Sleep Habits Questionnaire

The CSHQ Short Form comprises 33 items^20^ in 8 subscales specified for the following sleep-related issues: resistance to going to bed, difficulty falling asleep, sleep duration, anxiety during sleep, waking up during the night, parasomnias, breathing difficulties during sleep and daytime tiredness. The total score of this scale ranges from 33 to 99 with a cut-off value of 41 points. Higher scores were considered clinically significant, indicating more sleep problems. Nine subitems were assessed: inattention, aggressiveness, increased mobility/restlessness, non-compliance/disobedience, anxiety/fear/concerns, restlessness, sadness/unhappiness and physical complaints (eg, pain, fatigue and weakness). The Turkish version of the scale has been shown to have confirmed convergent and discriminant validity, as well as internal reliability (Cronbach’s alpha=0.78) for elementary school children.^15^

#### Epworth Sleepiness Scale for Children

The ESS-CHAD is an eight-item self-report questionnaire designed to evaluate the intensity of daytime sleepiness in various activities and settings that occur in the daily routines of children and adolescents.^21^ A higher score indicates a greater degree of daytime sleepiness. The Turkish version of the scale has been shown to have confirmed convergent and discriminant validity, as well as internal reliability (Cronbach’s alpha=0.74) for Turkish children aged 12–18 years.^16^

#### Pediatric Daytime Sleepiness Scale

The PDSS is a specialised tool designed to assess the level of daytime sleepiness in children and adolescents.^22^ It consists of eight items associated with sleep behaviour, with Likert-type responses. The total score ranges from 0 to 32 points. A positive correlation has been determined between the scores obtained by children and adolescents on the scale and their level of daytime sleepiness. The Turkish version of the scale has been shown to have confirmed convergent and discriminant validity as well as internal reliability for typically developed school students (Cronbach’s alpha=0.79).^17^

#### Pediatric Sleep Questionnaire

The PSQ was developed by Chervin *et al* to evaluate sleep-related breathing problems.^23^ The scale includes three subdivisions of Night-time and Bedtime Behaviours, Behaviours During the Day and Possible Problems and Attention Deficit and Hyperactivity Disorder (ADHD). The total score ranges between 0 and 70, with higher scores indicating the presence of sleep problems. The Turkish version of the scale has been shown to have confirmed convergent and discriminant validity, as well as internal reliability (Cronbach’s alpha=0.77) for children with ADHD aged 4–14 years.^18^

#### Sleep Disturbance Scale for Children

The SDSC assesses children’s sleep behaviours and is used to identify sleep disorders.^24^ The inventory has 24 items, providing a total score in the range of 24 to 120 points, with higher scores indicating a greater severity of acute sleep problems. The Turkish version of the scale has been shown to have confirmed convergent and discriminant validity as well as internal reliability (Cronbach’s alpha=0.81) for children between the ages of 6–15 years.^19^

### Statistical analysis

SAS V.9.4 software (Statistical Analysis Software V.9.4) was used for statistical analysis of the study data. Descriptive statistics were reported as mean±SD values for quantitative variables determined by measurement, and as number (n) and percentage (%) for qualitative variables determined by counting. First, the conformity of quantitative data to normal distribution was assessed using the Shapiro-Wilk test and assessment of skewness coefficients. Given that the test findings and skewness coefficients fell within the range of +3 to −3 for all variables, the data exhibited normal distribution, so parametric tests were employed for statistical analyses. The t-test was used for independent groups to compare variables between the two categories. Analysis of variance (F-test) was employed to identify differences between variables with three or more categories. χ² analysis was performed to determine associations between qualitative variables. Multiple receiver operating characteristic (ROC) analyses were performed to assess the precision of each index test. The threshold value was selected based on the Youden index, which was calculated by adding the sensitivity and specificity and subtracting 1. A t-test was used for independent groups to compare the variables between the two categories.

Cronbach’s alpha coefficient is classified in the categories of excellent (>0.90), good (>0.80), acceptable (>0.70), questionable (>0.60) and unacceptable (>0.5).^25^ A Cronbach’s alpha rating of ≥0.7 indicates an adequate level of reliability.^26^ The level of statistical significance was set at 0.05.

## Results

Evaluation was made of 155 children diagnosed with ASD with an average age of 9±3.69 years, and 75 typically developed peers with an average age of 9.07±4.25 years. The average body mass index was 18.1±4.88 for the children with ASD, and 20.6±4.77 for the typically developed peer group. In the ASD group, 84.5% of the children were male, and 15.5% were female. In both groups, 40% of the children were in preschool education. The sociodemographic characteristics of the participants are presented in table 1.

**Table 1:** Descriptive statistics of the sociodemographic data of the children

Parameters	ASD (n=155)	Typically developed peers (n=75)	P value	t
	X (SD)	X (SD)		
Age (years)	9.0 (3.69)	9.07 (4.25)	0.9	−0.13
Height (cm)	135.0 (22.0)	130.04 (28.0)	0.17	1.34
Weight (kg)	35.3 (20.2)	36.6 (18.8)	0.63	−0.47
BMI (kg/m^2^)	18.1 (4.88)	20.6 (4.77)	0.001*	−3.74
Gestational age (weeks)	38.3 (2.56)	38.5 (1.20)	0.46	−0.37
Birth weight (kg)	3.17 (7.17)	3.22 (2.55)	0.54	−0.61

	n (%)	n (%)	P value	X^2^
Gender				
Male	131 (84.5)	59 (78.7)	0.27	1.204
Female	24 (15.5)	16 (21.3)		

	n (%)	n (%)	P value	X^2^
Education level				
Preschool	63 (40.6)	30 (40)	0.77	1.1
Elementary	53 (34.1)	22 (23.34)		
Middle	24 (15.4)	13 (17.33)		
High	15 (9.6)	10 (13.3)		

*p<0.05.

ASD, autism spectrum disorder; BMI, body mass index; n, count; t, Student's t-test; X, mean.

The descriptive statistics, comparisons of discriminant validity and internal consistency values of the CSHQ, ESS-CHAD, PDSS, PSQ, and SDSC are presented in table 2. The results showed that all the scales assessed in the study demonstrated discriminant validity for sleep disturbance in individuals with ASD (p<0.05). The CSHQ had excellent Cronbach’s alpha (0.96). The ESS-CHAD was deemed questionably acceptable (0.67), the PDSS had a good level of validity (0.83), and the PSQ (0.94) and SDSC (0.91) demonstrated excellent internal consistency (table 2).

**Table 2:** Discriminative comparisons and Cronbach’s alpha coefficient values of each scale for sleep-wake disorders

	ASD (n=155)	Typically developed peers (n=75)	P value	Cronbach’s Alpha
	X (SD)	X (SD)		
CSHQ				
Bedtime resistance	15.2 (2.58)	7.1 (0.40)	0.001*	0.91
Sleep onset delay	2.2 (0.77)	1.0 (0.16)	0.001*	na
Sleep duration	6.2 (1.36)	3.1 (0.27)	0.001*	0.89
Sleep anxiety	7.9 (1.65)	4.1 (0.34)	0.001*	0.79
Night wakings	4.9 (1.20)	3.1 (0.41)	0.001*	0.63
Parasomnias	11.1 (2.13)	7.1 (0.29)	0.001*	0.71
Sleep disordered breathing	4.2 (1.23)	3.0 (0.00)	0.001*	0.55
Daytime sleepiness	15.1 (3.29)	6.2 (0.49)	0.001*	0.91
CSHQ-Total	60.3 (7.92)	31.7 (1.03)	0.001*	0.96
PSQ				
Night-time and bedtime behaviours	14.8 (5.45)	2.4 (1.87)	0.001*	0.92
Behaviours during the day and possible problems	6.2 (2.36)	2.3 (0.95)	0.001*	0.73
Attention deficit and hyperactivity	4.0 (1.50)	0.0 (0.00)	0.001*	0.85
PSQ-Total	73.7 (19.86)	66.1 (15.33)	0.003*	0.96
SDSC				
Disorders of initiating and maintaining sleep	16.7 (5.43)	9.1 (1.01)	0.001*	0.85
Sleep breathing disorders	4.5 (2.01)	3.1 (0.27)	0.001*	0.67
Disorders of arousal	5.1 (1.98)	3.3 (0.50)	0.001*	0.68
Sleep-wake transition disorders	8.7 (1.70)	4.3 (0.46)	0.001*	0.55
Disorders of excessive somnolence	8.2 (2.25)	5.5 (1.22)	0.001*	0.58
Sleep hyperhidrosis	3.4 (2.15)	2.3 (0.47)	0.001*	0.89
SDSC Total	46.6 (11.54)	27.6 (1.45)	0.001*	0.91
ESS-CHAD Total	5.6 (3.61)	1.0 (0.68)	0.001*	0.67
PDSS-Total	15.3 (3.43)	4.8 (0.67)	0.001*	0.83

* p<0.05.

ASD, autism spectrum disorder; CSHQ, Children’s Sleep Habits Questionnaire; ESS-CHAD, Epworth Sleepiness Scale for Children and Adolescents; n, count; na, not applicable; PDSS, Paediatric Daytime Sleepiness Scale; PSQ, Paediatric Sleep Questionnaire; SDSC, Sleep Disturbance Scale for Children; t, Student's t-test; X, mean.

**Figure 1:**
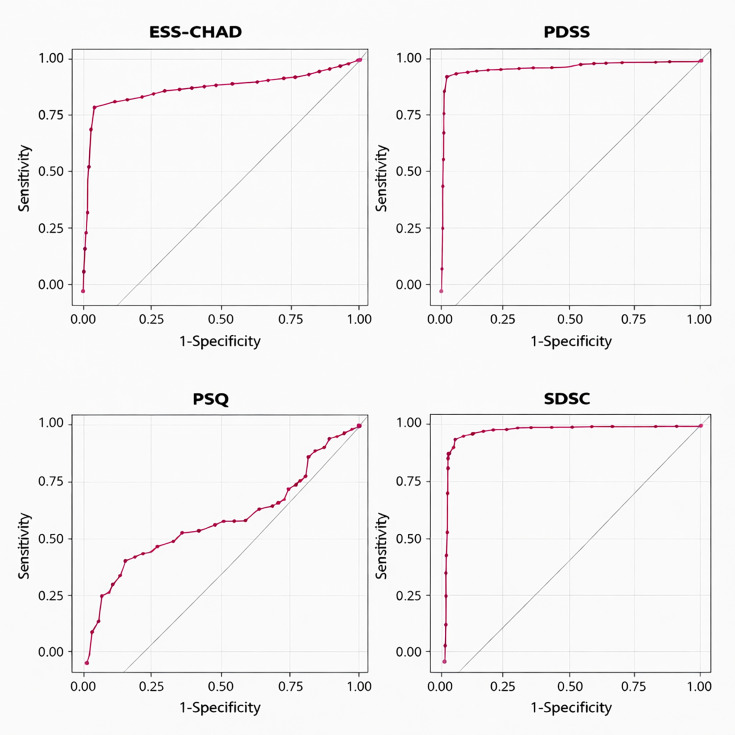
ROC curves of each scale based on the CSHQ cut-off value. CSHQ, Children’s Sleep Habits Questionnaire; ESS-CHAD, Epworth Sleepiness Scale for Children and Adolescents; PDSS, Paediatric Daytime Sleepiness Scale; PSQ, Paediatric Sleep Questionnaire; ROC, Receiver Operating Characteristic; SDSC, Sleep Disturbance Scale for Children.

The results of the ROC analysis according to the CSHQ cut-off values are shown in table 3 and figure 1. The AUC (area under the curve) values of the ESS-CHAD, PDSS, PSQ, and SDSC scales were 0.86, 0.99, 0.63 and 1, respectively (p<0.05). The positive predictive value (PPV) was 0.99 for the ESS-CHAD and PDSS, 1 for the PSQ and 0.86 for the SDSC. The negative predictive value (NPV) values of the ESS-CHAD, PDSS, PSQ and SDSC scales were 0.99, 0.7, 0.94 and 0.44, respectively.

**Table 3:** ROC analysis of each scale based on CSHQ cut-off value

Scale	AUC (95% CI)	Sensitivity (95% CI)	Specificity (95% CI)	PPV (95% CI)	NPV (95% CI)	Agreement (95% CI)
ESS-CHAD	0.86 (0.80 to 0.91)	0.79 (0.73 to 0.86)	0.99 (0.96 to 1.00)	0.99 (0.98 to 1.00)	0.99 (0.96 to 1.00)	0.99 (0.97 to 1.00)
PDSS	0.99 (0.97 to 1.00)	0.97 (0.94 to 0.99)	1.00 (1.00 to 1.00)	0.99 (0.98 to 1.00)	0.70 (0.61 to 0.79)	0.86 (0.80 to 0.90)
PSQ	0.63 (0.50 to 0.70)	0.47 (0.39 to 0.55)	0.85 (0.78 to 0.93)	1.00 (1.00 to 1.00)	0.94 (0.88 to 0.99)	0.98 (0.95 to 0.99)
SDSC	1.00 (0.99 to 1.00)	0.99 (0.98 to 1.00)	0.97 (0.96 to 1.00)	0.86 (0.80 to 0.94)	0.44 (0.36 to 0.52)	0.60 (0.53 to 0.66)

AUC, area under the curve; CSHQ, Children’s Sleep Habits Questionnaire; ESS-CHAD, Epworth Sleepiness Scale for Children and Adolescents; NPV, negative predictive value; PDSS, Paediatric Daytime Sleepiness Scale; PPV, positive predictive value; PSQ, Paediatric Sleep Questionnaire; ROC, receiver operating characteristic; SDSC, Sleep Disturbance Scale for Children.

## Discussion

Evaluations were made in this study of the sleep-wake characteristics of 155 children diagnosed with ASD aged 4–18 years, and 75 typically developed children. From the comparative analyses of various sleep and wakefulness scales, it was seen that children with ASD experienced more sleep disturbances than their typically developed peers. Employing the CSHQ, ESS-CHAD, PDSS, PSQ and SDSC assessment tools in combination may be considered promising for the assessment of sleep-wake disorders in this population.

Previous investigations have documented that a significant proportion of children with ASD experience persistent sleep difficulties.^27 28^ In a study conducted by Williams Buckley *et al* it was reported that the most common sleep problems experienced by this population included difficulties in initiating and maintaining sleep, frequent and often prolonged night awakenings, early morning awakenings, sleepiness during the day, and irregular sleep-wake hours.^29^ In another recent investigation, Chen *et al* reported that difficulty falling asleep was the most common sleep problem of children with ASD. It was also seen that children with ASD have difficulty maintaining sleep, frequent night awakenings, and sleep fragmentation.^28^ The current study results demonstrated that children with ASD experience significantly higher levels of bedtime resistance, delay in falling asleep, nocturnal awakenings, sleep duration, and daytime sleepiness than their typically developing peers. Specifically, bedtime resistance was seen to be 114% higher, delay in falling asleep 120% higher, nocturnal awakenings 58% higher, sleep duration 99% higher, daytime sleepiness 143% higher, and parasomnias were 56% higher in children with ASD. These problems may contribute to the poor sleep quality and increased daytime sleepiness in children with ASD. Therefore, it is important for clinicians to regularly assess sleep problems in children with ASD in clinical practice.

Actigraphy, polysomnography, and clinical assessment scales are commonly used to evaluate sleep disorders in children with ASD.^30 31^ Although actigraphy has been shown to have reliability and validity for measuring sleep in children with ASD,^31^ this method has the limitations of accessibility, high cost, and that it is time-consuming. Also, actigraphy may be sensitive to movement artefacts and may show reduced specificity in detecting wakefulness, particularly in children with ASD who often exhibit increased nocturnal movements. In clinical settings, scales based on parent reports provide practical information on sleep habits and the impact of sleep problems on daily life.^13 32^ The CSHQ, ESS-CHAD, PDSS, PSQ and SDSC were the scales used in the current study to evaluate sleep problems. To the best of our knowledge, as no previous study has examined the advantages and disadvantages of these scales for the clinical assessment of sleep problems in children with ASD, this is the first study to analyse these sleep scales in detail in this population.

The CSHQ has been used as a reliable tool to assess sleep problems in various studies. Cronbach’s alpha coefficients for the subscales of the CSHQ, such as bedtime resistance, sleep duration, sleep anxiety, nocturnal awakenings, parasomnias, sleep-disordered breathing and daytime sleepiness, have generally been found to be within acceptable ranges (0.36–0.93), although they vary between studies.^15 33 34^ In line with the literature, the CSHQ in different languages is considered an adaptable and reliable scale for the assessment of sleep problems in children.

Previous studies on paediatric populations with typical development, narcolepsy or cataplexy have demonstrated the validity of the ESS-CHAD.^16 21^ To the best of our knowledge, the discriminant validity and internal consistency of the ESS-CHAD have not been previously assessed in children with ASD. The current study results revealed that the ESS-CHAD has both discriminant validity and internal consistency in children with ASD.

Previous studies of adolescents with typical development and those examining different cultural adaptations have shown that the PDSS has good reliability.^17 35^ The findings of the current study were similar to those reported in the literature. Therefore, the PDSS is considered a valid tool for determining sleep problems in children with ASD. In studies examining the internal consistency of the PSQ, it has been observed that the scale scores generally had acceptable Cronbach’s alpha values.^36 37^ In the current study, the Cronbach alpha values of the PSQ were found to be adequate and excellent. These findings confirm that the PSQ demonstrates discriminant validity for identifying sleep problems in children with ASD.

The SDSC has demonstrated good reliability in various studies.^19 38^ The current study results showed that the SDSC has acceptable-high reliability, with Cronbach’s alpha values ranging between 0.55 and 0.90. These findings suggest that the SDSC is a valid tool for the assessment of sleep disturbances in individuals with ASD.

ROC analysis is commonly used to assess the efficacy of various scales employed in clinical settings to identify the presence of a condition, thereby playing a vital role in informing clinical decision-making for a range of diagnostic tools. This analytical technique is of great significance in the determination of the discrimination capabilities of different scales employed in clinical settings.^39 40^ To the best of our knowledge, there are no comprehensive ROC analysis studies in the literature on scales that assess sleep problems in children with ASD. This study is the first to show that the CSHQ and SDSC have similar levels of diagnostic accuracy in detecting sleep disorders. This suggests that the CSHQ and SDSC can be used as alternatives for detecting sleep disorders. Furthermore, the SDSC may be a good alternative for researchers who want to examine the effects of sleep disturbance on behavioural characteristics in children with ASD. The PSQ is characterised by a high PPV, especially in the assessment of sleep problems involving respiratory disorders. In contrast, the specificity of the PDSS and both the high NPV and agreement level of the ESS-CHAD emphasise the capacity of these scales to accurately discriminate individuals without sleep disorders. These results support that the ESS-CHAD, in particular, can be recommended for the rapid and effective assessment of sleep problems in children with ASD. In this context, the use of the PDSS or ESS-CHAD can be recommended if clinicians have limited time for the assessment phase.

In conclusion, the results of this study demonstrated that children with ASD experience significantly greater sleep-wake disturbances than their typically developing peers across all assessment tools examined. Each questionnaire exhibited distinct psychometric strengths, and the selection of the most appropriate instrument may depend on the specific clinical context and the primary sleep-related concerns reported by caregivers (online supplemental material 1). Rather than advocating for the routine use of all available instruments, the findings of this study suggest that clinicians may tailor their assessment approach based on clinical presentation and available resources. It is essential to incorporate sleep assessment into the clinical management of children with ASD, as sleep disturbances can significantly affect their daily functioning and developmental outcomes.

Despite the strengths of the study, an important limitation was that a population with a wide age range was analysed. Considering the diversity of sleep needs in different age groups, there is a need for further studies in more homogeneous age groups. In addition, the gender distribution in the ASD group was predominantly male, which reflects the known epidemiological pattern of ASD. Although gender was not included as a covariate in the ROC analyses, future studies may further explore potential gender-related differences in sleep characteristics among children with ASD. Also, considering the diversity of sleep needs and developmental characteristics across childhood and adolescence, the performance of sleep questionnaires may vary according to age. Caregiver-report scales such as the CSHQ may be more appropriate for younger children, whereas self-report instruments such as the ESS-CHAD may provide more reliable information in older children and adolescents who are able to report their own sleep experiences. Therefore, future studies examining more homogeneous age groups may help clarify potential age-related differences in the performance of sleep assessment tools. Additionally, future research incorporating long-term follow-up designs and examining the clinical utility of targeted instrument selection based on presenting symptoms and available resources may both strengthen the current findings and provide more practical guidance for clinicians working in resource-limited settings.

## Conclusions

Children with ASD tend to experience higher levels of sleep-wake disturbances than their typically developing peers, as demonstrated by the various scales used in this study to assess sleep-wake disorders. The CSHQ was found to have the closest discrimination and sensitivity in detecting sleep disturbances in children with ASD. According to the CSHQ, the PDSS is the scale that best distinguishes those who are not truly affected. The CSHQ also indicates that the scale showing the probability of being truly affected in the population is the PSQ and the scale showing the probability of being not affected is the ESS-CHAD. The need to use a range of assessment tools, including the CSHQ, ESS-CHAD, PDSS, PSQ and SDSC, as in the current study, comes to the fore given the prevalence of sleep difficulties in children. This approach is promising and important for the evaluation of sleep-wake disorders in children with ASD. It is essential to include the evaluation of sleep profiles in the treatment goals for children with ASD because these profiles can significantly affect the daily life and development of the child.

## Supplementary material

10.1136/bmjpo-2025-004097 online supplemental file 1

## Data Availability

Data may be obtained from a third party and are not publicly available.
