# Supplementary material for: Comparative accuracy of sleep disturbance questionnaires in children with autism spectrum disorder: a receiver operating characteristic study
Source: BMJ Paediatr Open. 2026 May 6;10(1):e004097. doi: 10.1136/bmjpo-2025-004097 (PMC13150884; doi:10.1136/bmjpo-2025-004097)
Supplement: online supplemental file 1 [file bmjpo-10-1-s001.docx]

**SUPPLEMENTARY MATERIAL 1**

**Clinical Decision Flowchart: Sleep Disturbance Assessment in Children with Autism Spectrum Disorder**

*A tiered, evidence-based instrument selection guide derived from ROC analysis of CSHQ, SDSC, ESS-CHAD, PDSS, and PSQ in a pediatric ASD cohort (n=155).*

*From:* Yüksel B, Doğan M, Kara K, Kaya Kara Ö. *How should we choose questionnaires to evaluate sleep disturbances in children with autism spectrum disorder for their clinical usefulness? A receiver operative characteristics study.*

| ■ Tier 1 – Comprehensive Screen | ■ Tier 2 – Broad Multidomain | ■ Tier 3 – Rapid / Time-limited | ■ Tier 4 – Targeted / Specialist |
| --- | --- | --- | --- |

| **Child with Confirmed ASD Diagnosis, Ages 4–18** |
| --- |

**↓**

| **Step 1: Is a sleep concern present or routine screening due?**  *Caregiver report · Clinical observation · Behavioral worsening* |
| --- |

**↓**

*YES → Proceed to Tier Selection*

| ◆ **Step 2: Select Instrument Tier**  *Based on clinical context & time available* |
| --- |

**↓ ↓ ↓ ↓**

| **TIER 1 · FIRST-LINE COMPREHENSIVE**  **CSHQ**  33-item, 8 subscales. Best overall diagnostic accuracy. Preferred for initial comprehensive evaluation of behavioral sleep habits.  AUC (Reference): **Gold Standard**  Cronbach α: **0.96 ★**  Cutoff: **≥ 41**  Best for: **General screen** | **TIER 2 · ALTERNATIVE BROAD SCREEN**  **SDSC**  24-item multidomain tool. Equivalent accuracy to CSHQ. Use when behavioral focus of CSHQ is less relevant.  AUC: **1.00**  Sensitivity: **0.99**  Specificity: **0.97**  Cronbach α: **0.91** | **TIER 3 · RAPID / TIME-LIMITED**  **ESS-CHAD · PDSS**  8-item scales. Use when clinical time is limited. ESS-CHAD for rapid follow-up; PDSS to rule-out sleepiness disorders.  ESS-CHAD AUC: **0.86**  ESS-CHAD Agreement: **0.99**  PDSS AUC: **0.99**  PDSS NPV: **0.70** | **TIER 4 · TARGETED / SPECIALIST**  **PSQ**  Use when sleep-disordered breathing is suspected. Highest PPV — confirms true positives. Not recommended as first-line screen.  AUC: **0.63**  PPV: **1.00 ★**  NPV: **0.94**  Cronbach α: **0.94** |
| --- | --- | --- | --- |

**↓**

*Interpret score against validated cut-off*

| **✓ Positive Screen**  Score ≥ cutoff or clinically elevated  *→ Document, initiate sleep intervention, consider referral* | **✗ Negative / Borderline**  Score below cutoff  *→ Add ESS-CHAD for rapid confirmation; re-screen in 3–6 months* |
| --- | --- |

**Instrument Performance Reference — ROC Analysis Summary**

| **Instrument** | **Items** | **Primary Strength** | **AUC** | **Sensitivity** | **Specificity** | **PPV** | **NPV** | **α** | **Recommended Use** |
| --- | --- | --- | --- | --- | --- | --- | --- | --- | --- |
| **CSHQ** | 33 | Overall accuracy + 8 subscales | **Reference** | — | — | — | — | 0.96 | **Tier 1 · First-line** |
| **SDSC** | 24 | Multidomain, equivalent to CSHQ | 1.00 | 0.99 | 0.97 | 0.86 | 0.44 | 0.91 | **Tier 2 · Alternative** |
| **PDSS** | 8 | High sensitivity, rule-in | 0.99 | 0.97 | 1.00 | 0.99 | 0.70 | 0.83 | **Tier 3 · Rapid rule-in** |
| **ESS-CHAD** | 8 | High agreement, fast | 0.86 | 0.79 | 0.99 | 0.99 | 0.99 | 0.67 | **Tier 3 · Rapid follow-up** |
| **PSQ** | 70 | Highest PPV (breathing focus) | 0.63 | 0.47 | 0.85 | 1.00 | 0.94 | 0.94 | **Tier 4 · Targeted SDB** |

| **Clinical Note:** All five instruments demonstrated discriminant validity in children with ASD (p<0.05). For comprehensive assessment, combined use is recommended.  CSHQ cutoff ≥ 41 · PDSS: higher scores = greater daytime sleepiness · ESS-CHAD: higher scores = greater daytime sleepiness · PSQ: higher scores = more sleep problems · SDSC: range 24–120.  *SDB = Sleep-disordered breathing · AUC = Area Under the Curve · PPV = Positive Predictive Value · NPV = Negative Predictive Value · α = Cronbach’s Alpha* |
| --- |
