# Supplementary material for: Comparative accuracy of sleep disturbance questionnaires in children with autism spectrum disorder: a receiver operating characteristic study
Source: BMJ Paediatr Open. 2026 May 6;10(1):e004097. doi: 10.1136/bmjpo-2025-004097 (PMC13150884; doi:10.1136/bmjpo-2025-004097)
Supplement: Supplementary file 2 [file bmjpo-2025-004097.reviewer_comments.pdf]

PEER REVIEW HISTORY

BMJ Paediatrics Open publishes all reviews undertaken for accepted manuscripts. Reviewers are asked to complete a checklist review form and are provided with free text boxes to elaborate on their assessment. These free text comments are reproduced below.

ARTICLE DETAILS

**TITLE (PROVISIONAL)**	Comparative accuracy of sleep disturbance questionnaires in children with autism spectrum disorder: A receiver operative characteristics study
**AUTHORS**	Yüksel, Batuhan<br>Dogan, Mert<br>Kara, Koray<br>Kaya Kara, Ozgun

VERSION 1 – REVIEW

**REVIEWER NAME**	Sahin, Sedef
**REVIEWER AFFILIATION**	Hacettepe University, Department of Occupational Therapy
**REVIEWER CONFLICT OF INTEREST**	No Conflict of Interest
**AI DISCLOSURE**	No, I have not used AI
**DATE REVIEW RETURNED**	30-Oct-2025

**GENERAL COMMENTS**	The manuscript entitled 'Comparative accuracy of sleep disturbance questionnaires in children with autism spectrum disorder: A receiver operative characteristics study' examines the diagnostic accuracy of three commonly used parent-reported sleep disturbance questionnaires (the Children's Sleep Habits Questionnaire [CSHQ], Sleep Disturbance Scale for Children [SDSC], and Pediatric Sleep Questionnaire [PSQ]) against actigraphy as the reference standard in children with autism spectrum disorder (ASD). Using ROC analysis, the authors aim to determine optimal cut-off scores and evaluate each tool’s sensitivity and specificity. The study addresses a clinically relevant gap in pediatric sleep assessment, especially within neurodevelopmental populations.<br>I congratulate you on your study, which will make a valuable contribution to the field. Some of my suggestions for your study are as follows<br>1- Recruitment from a single tertiary center may introduce bias. It would be valuable to clarify inclusion/exclusion criteria and whether children with intellectual disability or medication use were excluded.<br>2- Although actigraphy is widely used, it is not a perfect gold standard for sleep assessment in ASD populations. Please acknowledge its limitations (e.g., sensitivity to movement artifacts and reduced specificity for wake detection).<br>3- The discussion could elaborate more on how clinicians should choose between questionnaires in practice. For instance, under what circumstances should SDSC be preferred over CSHQ or PSQ?<br>4- Table 3 contains dense numeric data that could be visually enhanced using shading or simplified footnotes to improve readability.

**REVIEWER NAME**	Gulati, Sheffali

**REVIEWER AFFILIATION**	All India Institute of Medical Sciences New Delhi, Child NeurologyDivision, Pediatrics
**REVIEWER CONFLICT OF INTEREST**	No Conflict of Interest
**AI DISCLOSURE**	No, I have not used AI
**DATE REVIEW RETURNED**	26-Feb-2026

**GENERAL COMMENTS**	The authors have conducted a highly relevant and timely study that addresses a significant clinical gap, the need for validated accessible tools to identify sleep disturbances in children with autism. By providing a rigorous comparative analysis of five different scales using ROC curve methodology, this research offers valuable evidence-based guidance that can directly improve diagnostic accuracy and quality of life for this vulnerable population.<br>However, some major points that needs to be addressed are -<br>• While the introduction successfully establishes the high prevalence of sleep disturbances in children with ASD, it could more explicitly detail why these five specific questionnaires were chosen for comparison over others. A brief sentence describing the unique features or theoretical frameworks of each tool would better justify their selection for this comparative analysis.<br>• The text mentions that clinicians face challenges in selecting the most appropriate tool. The introduction would be enhanced by identifying specific gaps in previous literature regarding the "discriminatory performance" of these tools in Turkish populations or specifically within the wide 4–18 age range used in this study.<br>• The participants were recruited from a regional referral center. Providing more detail on the recruitment process—such as whether they were consecutive patients or recruited via outreach—would help readers assess potential selection bias and the generalizability of the findings.<br>• The exclusion criteria for the typically developing group included "the presence of any sleep problems". It would be helpful to clarify if this was determined by a clinical screening or purely by parent report, as this could impact the baseline comparison with the ASD group who were diagnosed by a specialist.<br>• Table 1 and the text note a significant gender imbalance in the ASD group (84.5% male) compared to the control group. A brief comment in the Results or Discussion addressing whether gender was considered as a covariate in the ROC analysis would add depth, as sleep patterns can vary by gender in neurodevelopmental disorders.<br>• The results present a high volume of statistical data (AUC, PPV, NPV, sensitivity, specificity) for multiple scales. Presenting a simplified summary table or a single sentence that ranks the tools based on a "clinical utility index" (balancing accuracy with ease of use) would make the findings more accessible for practitioners.<br>• The authors correctly identify the wide age range (4–18 years) as a limitation. The Discussion could be further enriched by briefly mentioning how neurodevelopmental changes from early childhood to late adolescence might specifically affect the performance of "caregiver-report" scales like the CSHQ versus "self-report" scales like the ESS-CHAD.<br>• The conclusion emphasizes the need for a range of tools. Providing a more specific "decision-making flowchart" or a tiered recommendation (e.g., "Use CSHQ for initial screening and PDSS for rapid follow-up") would transform the academic findings into highly actionable clinical guidance.

VERSION 1 – AUTHOR RESPONSE

RESPONSE TO REVIEWER'S COMMENTS

Reviewer1

Comment: The manuscript entitled 'Comparative accuracy of sleep disturbance questionnaires in children with autism spectrum disorder: A receiver operative characteristics study' examines the diagnostic accuracy of three commonly used parent-reported sleep disturbance questionnaires (the Children's Sleep Habits Questionnaire [CSHQ], Sleep Disturbance Scale for Children [SDSC], and Pediatric Sleep Questionnaire [PSQ]) against actigraphy as the reference standard in children with autism spectrum disorder (ASD). Using ROC analysis, the authors aim to determine optimal cut-off scores and evaluate each tool's sensitivity and specificity. The study addresses a clinically relevant gap in pediatric sleep assessment, especially within neurodevelopmental populations.
I congratulate you on your study, which will make a valuable contribution to the field. Some of my suggestions for your study are as follows:
Response: Thank you very much for your careful evaluation of our manuscript and for your positive and encouraging comments. We sincerely appreciate your recognition of the clinical relevance of our study and its potential contribution to the assessment of sleep disturbances in children with autism spectrum disorder (ASD). We are grateful for your constructive suggestions, which have helped us to further improve the clarity and scientific quality of the manuscript. In response to your comments, we have carefully revised the manuscript and provided detailed point-by-point responses below. All relevant sections of the manuscript have been updated accordingly to address the issues raised.
Comment-1 : Recruitment from a single tertiary center may introduce bias. It would be valuable to clarify inclusion/exclusion criteria and whether children with intellectual disability or medication use were excluded.
Response 1: Thank you for this valuable comment. To improve clarity, we have further specified the inclusion and exclusion criteria in the Methods – Participants section. Information regarding comorbid conditions, intellectual disability, and medication use has been clarified in the revised manuscript.
"The eligibility criteria for children with ASD were as follows: (1) a confirmed diagnosis of ASD by a child and adolescent psychiatrist according to DSM-5 criteria, (2) an age range of 4–18 years, and (3) willingness to participate in the study. Children with comorbid neurological or genetic disorders that could independently affect sleep patterns (e.g., cerebral palsy or genetic syndromes) were excluded. Information regarding intellectual disability and medication use was obtained from medical records and caregiver reports. The inclusion criteria for typically developing children were: (1) no diagnosis of neurological or psychiatric disorders and (2) age between 4 and 18 years. The exclusion criteria for typically developing children were (1) unwillingness to participate in the study and (2) the presence of reported sleep problems."

Comment 2: Although actigraphy is widely used, it is not a perfect gold standard for sleep assessment in ASD populations. Please acknowledge its limitations (e.g., sensitivity to movement artifacts and reduced specificity for wake detection).
Response 2: Thank you for this valuable comment. We would like to clarify that actigraphy was not used as a reference standard in the present study. The aim of this study was to compare the diagnostic performance of commonly used sleep questionnaires in children with ASD using ROC analysis based on questionnaire cut-off values.

However, we agree that objective sleep assessment tools such as actigraphy have important methodological limitations in ASD populations. To address this point, we have expanded the Discussion section to acknowledge the limitations of actigraphy, including its sensitivity to movement artifacts and reduced specificity for wake detection.
"….this method has the limitations of accessibility, high cost, and that it is time-consuming. Also, actigraphy may be sensitive to movement artifacts and may show reduced specificity in detecting

wakefulness, particularly in children with ASD who often exhibit increased nocturnal movements. In clinical settings, scales based on parent reports provide practical information on sleep habits and the impact of sleep problems on daily life…"
Comment 3: The discussion could elaborate more on how clinicians should choose between questionnaires in practice. For instance, under what circumstances should SDSC be preferred over CSHQ or PSQ?
Response 3: Based on your valuable feedback, we revised the paragraph as below:
"…This study compared the CSHQ, ESS-CHAD, PDSS, PSQ, and SDSC assessment tools and found each to be reliable in terms of various features. From a clinical perspective, these tools may serve different purposes. The CSHQ may be particularly useful for a comprehensive evaluation of general sleep habits and behavioral sleep problems, whereas the SDSC provides a broader assessment of multiple domains of sleep disturbances. The PSQ may be particularly informative when symptoms related to sleep-disordered breathing are suspected. Therefore, the selection of questionnaires may depend on the specific clinical context and the primary sleep-related concerns reported by caregivers. The findings of this study are expected to contribute to the existing literature and provide guidance for clinicians and researchers in selecting appropriate assessment tools…"

Comment 4: Table 3 contains dense numeric data that could be visually enhanced using shading or simplified footnotes to improve readability.

Response 4: Thank you for this helpful suggestion. To improve readability, the presentation of Table 3 has been revised. The table formatting has been simplified and explanatory notes have been clarified to make the information easier to interpret.

Reviewer: 2

Dr. Sheffali Gulati, All India Institute of Medical Sciences New Delhi
Comments to the Author

General Comment: The authors have conducted a highly relevant and timely study that addresses a significant clinical gap, the need for validated accessible tools to identify sleep disturbances in children with autism. By providing a rigorous comparative analysis of five different scales using ROC curve methodology, this research offers valuable evidence-based guidance that can directly improve diagnostic accuracy and quality of life for this vulnerable population.

Response: We sincerely thank the reviewer for the positive and encouraging comments. We are pleased that the reviewer recognizes the clinical relevance of our study and its potential contribution to improving the assessment of sleep disturbances in children with autism spectrum disorder. We appreciate the reviewer's evaluation of our work and the recognition of its potential clinical implications.

Comment 1: While the introduction successfully establishes the high prevalence of sleep disturbances in children with ASD, it could more explicitly detail why these five specific questionnaires were chosen for comparison over others. A brief sentence describing the unique features or theoretical frameworks of each tool would better justify their selection for this comparative analysis.
Response 1: Thank you for this helpful suggestion. We agree that further clarification regarding the rationale for selecting these questionnaires would strengthen the introduction. Therefore, we have added a brief explanation describing the main characteristics and clinical focus of each questionnaire (CSHQ, ESS-CHAD, PDSS, PSQ, and SDSC) to better justify their inclusion in this comparative analysis.
"These questionnaires were selected because they assess complementary dimensions of sleep and

wakefulness in pediatric populations. The CSHQ primarily evaluates behavioral sleep habits and sleep-related difficulties, the ESS-CHAD and PDSS focus on daytime sleepiness, the PSQ assesses sleep-related breathing symptoms and associated behavioral problems, and the SDSC provides a multidimensional assessment of different domains of sleep disturbances.”

Comment 2: The text mentions that clinicians face challenges in selecting the most appropriate tool. The introduction would be enhanced by identifying specific gaps in previous literature regarding the "discriminatory performance" of these tools in Turkish populations or specifically within the wide 4–18 age range used in this study.

Response 2: We thank the reviewer for this constructive suggestion. We agree that a clearer description of the existing gaps in the literature would strengthen the rationale for the study. Accordingly, the Introduction has been revised to emphasize the limited evidence regarding the comparative discriminatory performance of commonly used sleep questionnaires in Turkish pediatric populations and across the wide age range of 4–18 years. This addition clarifies the clinical and methodological motivation for the present study.
The likelihood …... These questionnaires were selected because they assess complementary dimensions of sleep and wakefulness in pediatric populations. The CSHQ primarily evaluates behavioral sleep habits and sleep-related difficulties, the ESS-CHAD and PDSS focus on daytime sleepiness, the PSQ assesses sleep-related breathing symptoms and associated behavioral problems, and the SDSC provides a multidimensional assessment of different domains of sleep disturbances. Most previous studies have focused on the validation or psychometric properties of individual instruments rather than evaluating their relative diagnostic performance 17-21. In addition, comparative evidence regarding the discriminatory performance of commonly used sleep questionnaires remains limited in Turkish pediatric populations, particularly across the wide developmental age range of 4–18 years. Therefore, the aim of this study was to investigate the comparative effectiveness of several widely used sleep and wakefulness scales in children with autism spectrum disorder. “

Comment 3: The participants were recruited from a regional referral center. Providing more detail on the recruitment process—such as whether they were consecutive patients or recruited via outreach—would help readers assess potential selection bias and the generalizability of the findings.

Response 3: We thank the reviewer for this helpful comment. To improve transparency regarding the recruitment process, we have clarified in the Methods section how participants were enrolled from the regional referral center.
“This study was conducted in the Department of Child and Adolescent Psychiatry at the University of Health Sciences Research and Training Hospital, Antalya, Türkiye, which serves as a regional referral center for children and adolescents with developmental and behavioral disorders. Participants were recruited from children presenting to this clinic during the study period who met the eligibility criteria.”
Comment 4: The exclusion criteria for the typically developing group included "the presence of any sleep problems". It would be helpful to clarify if this was determined by a clinical screening or purely by parent report, as this could impact the baseline comparison with the ASD group who were diagnosed by a specialist.

Response 4: We thank the reviewer for this important observation. To clarify this point, we have revised the Methods section to specify how sleep problems were identified in the typically developing group. The absence of sleep problems was determined based on caregiver reports obtained during the screening process and review of medical history.
“The exclusion criteria for typically developing children were (1) not willing to participate in the study and (2) the presence of any sleep problems, as determined based on caregiver reports obtained during the screening process and review of medical history.”

Comment 5: Table 1 and the text note a significant gender imbalance in the ASD group (84.5% male) compared to the control group. A brief comment in the Results or Discussion addressing whether gender was considered as a covariate in the ROC analysis would add depth, as sleep patterns can vary by gender in neurodevelopmental disorders.

Response 5: We thank the reviewer for this insightful comment. Gender was not included as a covariate in the ROC analysis, as the primary aim of the study was to evaluate the discriminatory performance of the sleep questionnaires at the scale level. However, we agree that the observed gender imbalance should be acknowledged when interpreting the findings. Therefore, we have added a statement in the Discussion section noting that the gender distribution reflects the well-established higher prevalence of ASD in males and that future studies may further explore potential gender-related differences in sleep characteristics.
"Despite the strengths of the study, an important limitation was that a population with a wide age range was analyzed. Considering the diversity of sleep needs in different age groups, there is a need for further studies in more homogeneous age groups. In addition, the gender distribution in the ASD group was predominantly male, which reflects the known epidemiological pattern of ASD. Although gender was not included as a covariate in the ROC analyses, future studies may further explore potential gender-related differences in sleep characteristics among children with ASD. ….."
Comment 6: The results present a high volume of statistical data (AUC, PPV, NPV, sensitivity, specificity) for multiple scales. Presenting a simplified summary table or a single sentence that ranks the tools based on a "clinical utility index" (balancing accuracy with ease of use) would make the findings more accessible for practitioners.

Response 6: We thank the reviewer for this helpful suggestion. To improve the clinical interpretability of the findings, a brief summary paragraph has been added to the Discussion section highlighting the potential clinical utility of the evaluated questionnaires.

In conclusion, the results of this study demonstrated that the sleep-wake disturbances experienced by children with ASD were consistently higher than those of their typically developed peers across all scales used. Accelerating global interest in sleep disorder research is crucial to be able to assess and address both the short- and long-term consequences of emerging issues. This study compared the CSHQ, ESS-CHAD, PDSS, PSQ, and SDSC assessment tools and found each to be reliable in terms of various features. From a clinical perspective, these tools may serve different purposes. The CSHQ may be particularly useful for a comprehensive evaluation of general sleep habits and behavioral sleep problems, whereas the SDSC provides a broader assessment of multiple domains of sleep disturbances. The PSQ may be particularly informative when symptoms related to sleep-disordered breathing are suspected. Therefore, the selection of questionnaires may depend on the specific clinical context and the primary sleep-related concerns reported by caregivers. The findings of this study are expected to contribute to the existing literature and provide guidance for clinicians and researchers in selecting appropriate assessment tools.

Comment 7: The authors correctly identify the wide age range (4–18 years) as a limitation. The Discussion could be further enriched by briefly mentioning how neurodevelopmental changes from early childhood to late adolescence might specifically affect the performance of "caregiver-report" scales like the CSHQ versus "self-report" scales like the ESS-CHAD.

Response 7: We thank the reviewer for this insightful suggestion. We agree that the wide developmental age range may influence the performance of different types of questionnaires. To address this point, we have expanded the Discussion section to briefly discuss how neurodevelopmental differences across childhood and adolescence may affect the use of caregiver-report and self-report sleep assessment tools.
…. Furthermore, developmental differences across childhood and adolescence may influence the

performance of sleep questionnaires. Caregiver-report scales such as the CSHQ may be more appropriate for younger children, whereas self-report instruments such as the ESS-CHAD may provide more reliable information in older children and adolescents who are able to report their own sleep experiences. Therefore, future studies examining more homogeneous age groups may help clarify potential age-related differences in the performance of sleep assessment tools. Future studies with long-term follow-up are also recommended to support the findings of this study….”
Comment 8: The conclusion emphasizes the need for a range of tools. Providing a more specific "decision-making flowchart" or a tiered recommendation (e.g., "Use CSHQ for initial screening and PDSS for rapid follow-up") would transform the academic findings into highly actionable clinical guidance.

Response 8: We thank the reviewer for this valuable suggestion. To enhance the clinical applicability of the findings, we have developed a schematic decision-making flowchart summarizing the potential clinical use of the evaluated questionnaires. This flowchart has been provided as Supplementary Material 1.
